# Comparison of *Helicobacter pylori* positive and negative gastric cancer via multi-omics analysis

**DOI:** 10.1128/mbio.01531-23

**Published:** 2023-10-17

**Authors:** Fumei Shang, Yinghao Cao, Lixin Wan, Zhonghai Ren, Xinghao Wang, Mudan Huang, Yingyun Guo

**Affiliations:** 1 Department of Medical Oncology, Nanyang Central Hospital, Nanyang, Henan, China; 2 Department of Digestive Surgical Oncology, Cancer Center, Union Hospital, Tongji Medical College, Huazhong University of Science and Technology, Wuhan, China; 3 Cancer Center, Union Hospital, Tongji Medical College, Huazhong University of Science and Technology, Wuhan, China; 4 Department of Radiation Oncology, The Third Affiliated Hospital of Shenzhen University (Shenzhen Luohu People's Hospital), Shenzhen, Guangdong, China; 5 Department of Gastroenterology, Renmin Hospital of Wuhan University, Wuhan, China; College of Veterinary Medicine, Cornell University, Ithaca, New York, USA

**Keywords:** gastric cancer, genetic analysis, *Helicobacter pylori*, untargeted metabolomics, dysbiosis

## Abstract

*Helicobacter pylori* (*H. pylori*) has been regarded as a definite carcinogenic bacterium for gastric cancer (GC). This multi-omics research was designed to investigate the genetic, microbial, and metabolic changes of GC patients when they are infected with *H. pylori*. We first mined The Cancer Genome Atlas Stomach Adenocarcinoma (STAD) data to identify the key genes and critical pathways in *H. pylori*-positive individuals with GC compared to *H. pylori*-negative individuals with GC. Then, fresh stool samples were collected from GC individuals screened for eligibility, and we analyzed the microbial changes and metabolite alterations between *H. pylori*-positive and *H. pylori*-negative GC individuals. Finally, we tried to explore the interaction between key gut flora and metabolite changes in GC patients infected with *H. pylori*. We identified three genes (GCG, APOA1, and IGFBP1) with significant relevance to *H. pylori* infection, and the survival monogram based on the three *H*. *pylori*-related genes showed good predictive ability for overall survival among GC individuals. 16S rRNA sequencing showed that the abundance of *Escherichia-Shigella, Bacteroides, Enterococcus*, *and Lactobacillus* was upregulated in GC cases with *H. pylori* at the level of genus. There exists a great difference in alpha and beta diversity between *H. pylori* group and non-*H*. *pylori* group. The untargeted metabolome analysis identified 295 significant fecal metabolites, and the levels of penitrem E, auberganol, stercobilinogen, and lys thr are upregulated in the *H. pylori* group. Finally, correlation analysis showed that there exists a significant correlation between the fecal metabolites and gut bacterial strains. This is the first clinical research to investigate the difference between GC patients with *H. pylori* and GC patients without *H. pylori* via multi-omics analysis. 16S rRNA sequencing along with untargeted metabolomics demonstrated decreased microbial diversity and metabolic dysregulation in gastric carcinoma individuals with *H. pylori* infection.

**IMPORTANCE:**

This is the first clinical research to systematically expound the difference between gastric cancer (GC) individuals with *Helicobacter pylori* and GC individuals without *H. pylori* from the perspective of multi-omics. This clinical study identified significant genes, microbes, and fecal metabolites, which exhibited nice power for differentiating GC individuals with *H. pylori* infection from GC individuals without *H. pylori* infection. This study provides a crucial basis for a better understanding of eradication therapy among the GC population.

## INTRODUCTION

Gastric cancer (GC) is one of the most common malignant lesions occurring in the digestive tract (1, 2), which has become a major health problem in China. The first-choice treatment for early GC is resection under endoscopy, while non-early operable GC should be treated with surgical resection. Advanced GC with remote metastasis should be treated with chemoradiotherapy or targeted therapy (3). The occurrence of GC is related to multiple factors, including high salt intake, genetic susceptibility, and dysbiosis (4). With the advent of novel technologies for detecting the microbes of the stomach, it is possible to gain better insight into the gastric microbiota. Colonization of *H. pylori* significantly impacts the microecology of the stomach, which in turn affects the colonic microbiota changes (5). Recent pre-clinical studies focusing on gastric microbiota demonstrated that GC patients exhibited reduced microbial diversity and abnormal bacterial interactions (6, 7). The predominant microbiota in GC revealed by a meta-analysis are *Parvimonas, Veillonella, Prevotella, Fusobacterium*, and *Peptostreptococcus* (8), which also promote the development and progression of GC. By contrast, the commonly reported commensals in normal gastric tissues include *Firmicutes* and *Streptococcus* (9). The close interactions between *Helicobacter pylori* and other non-*H*. *pylori* microbes may be also involved in the carcinogenesis and progression of GC (7). The tumor-associated bacteria occupy an important role in the initiation and progression of GC (10). Among them, *H. pylori* infection has been identified as a major risk factor for GC.

*H. pylori*, a Gram-negative bacterium, belongs to the *Campylobacterota* phylum, which is the predominant microbiota in the stomach after its infection (11). Infection with *H. pylori*, an aetiological agent in GC, is a serious public health concern all over the world. According to a recent meta-analysis (12), the prevalence of *H. pylori* infection was relatively stable between 1991 and 2010 but dropped sharply during the recent decade. Evidence has identified CagA as a promoting oncoprotein in GC, and infection with *H. pylori* harboring CagA protein is linked with a high degree of gastric inflammation (13). *H. pylori* stimulates the accumulation of neutrophils and lymphocytes in gastric mucosa with the production of inflammatory cytokines and reactive oxygen species, which further cause dysbiosis and disruption of gastric epithelial function (14, 15). Infiltration of inflammatory cells also contributes to cell proliferation and gastric carcinogenesis (16). Quite a few clinical studies proved that infection of *H. pylori* is correlated with a less favorable prognosis in individuals with GC. While we know nothing about the genetic profiling, alterations of tumor-associated bacteria, and metabolic changes of GC patients when they are infected with *H. pylori*. Elucidation of the exact mechanism of *H. pylori* infection could provide an important reference for the eradication of *H. pylori*.

Investigation of genetic profiling, microbiota alterations, and metabolic changes based on a prospective study design may help illuminate the role of *H. pylori* in the carcinogenesis and progression of GC. To fill the gap in the literature, we first mined The Cancer Genome Atlas Stomach Adenocarcinoma (TCGA-STAD) data to identify the key genes related to *H. pylori* infection and critical pathways in GC. Then, we prospectively collected the fresh stool samples from 51 cases of GC individuals and analyzed the microbial alterations and metabolic changes between *H. pylori*-positive and *H. pylori*-negative GC individuals via the 16S rRNA sequencing technique and untargeted metabolomics. Finally, we attempted to explore the interaction between key flora and metabolite changes in GC patients infected with *H. pylori*. Our study aimed to gain insights into the genetic, microbiota, and metabolic changes of GC patients infected with *H. pylori*, laying the foundation for the eradication of *H. pylori* and improving the prognosis of GC individuals.

## RESULTS

### Significant genes with *H. pylori* and enrichment analysis

**Fig 1:**
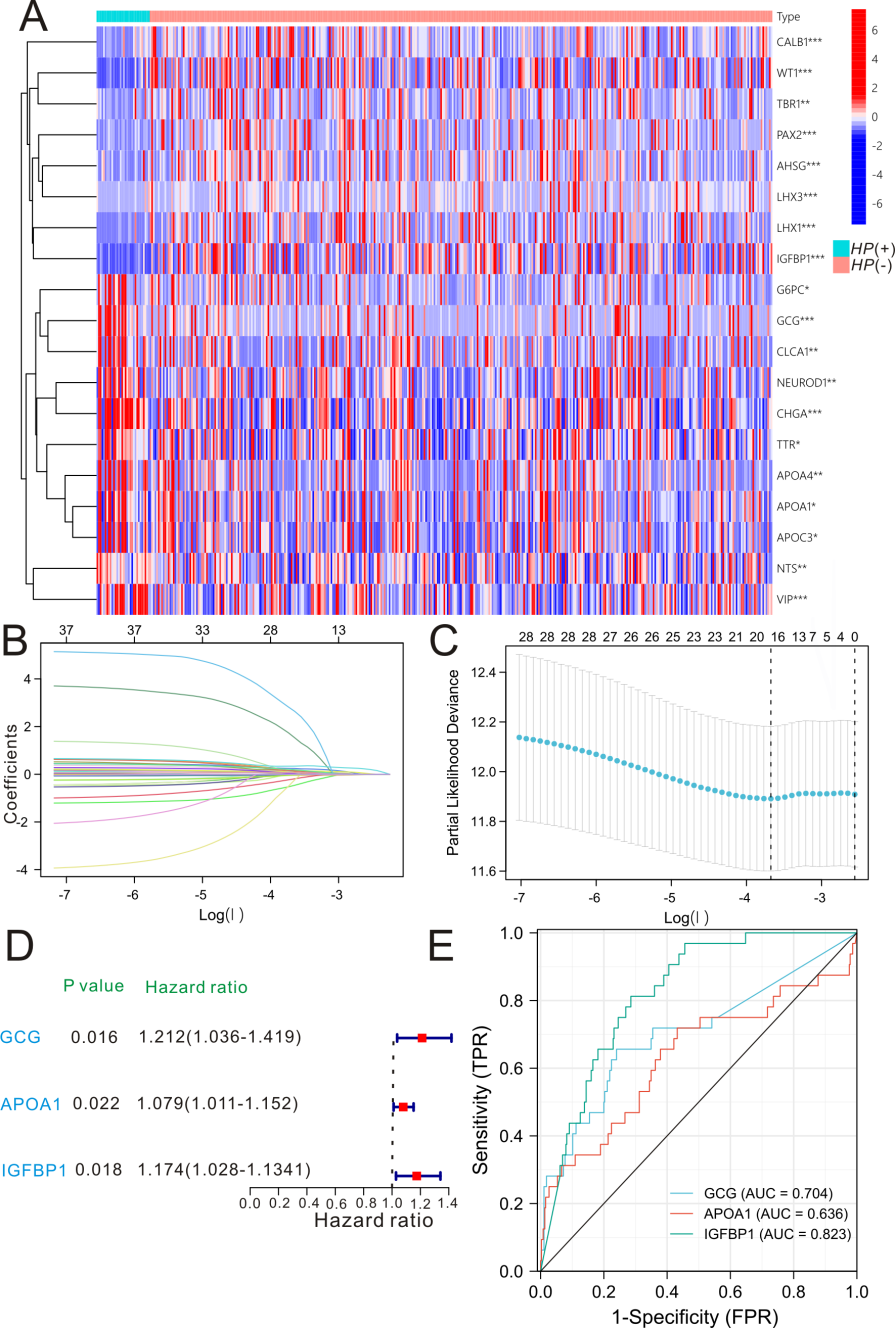
Selection process of *H. pylori*-related genes in gastric cancer. (**A)** Differentially expressed genes in gastric cancer individuals with *H. pylori* and without *H. pylori* revealed by heat map. (**B and C)** LASSO Cox for the selection of significant *H. pylori*-related genes in gastric cancer. (**D)** Univariate Cox regression of the three *H*. *pylori*-related genes in gastric cancer. (**E)** ROC curve of the three *H*. *pylori*-related genes for predicting the overall survival in gastric cancer individuals.

**Fig 2:**
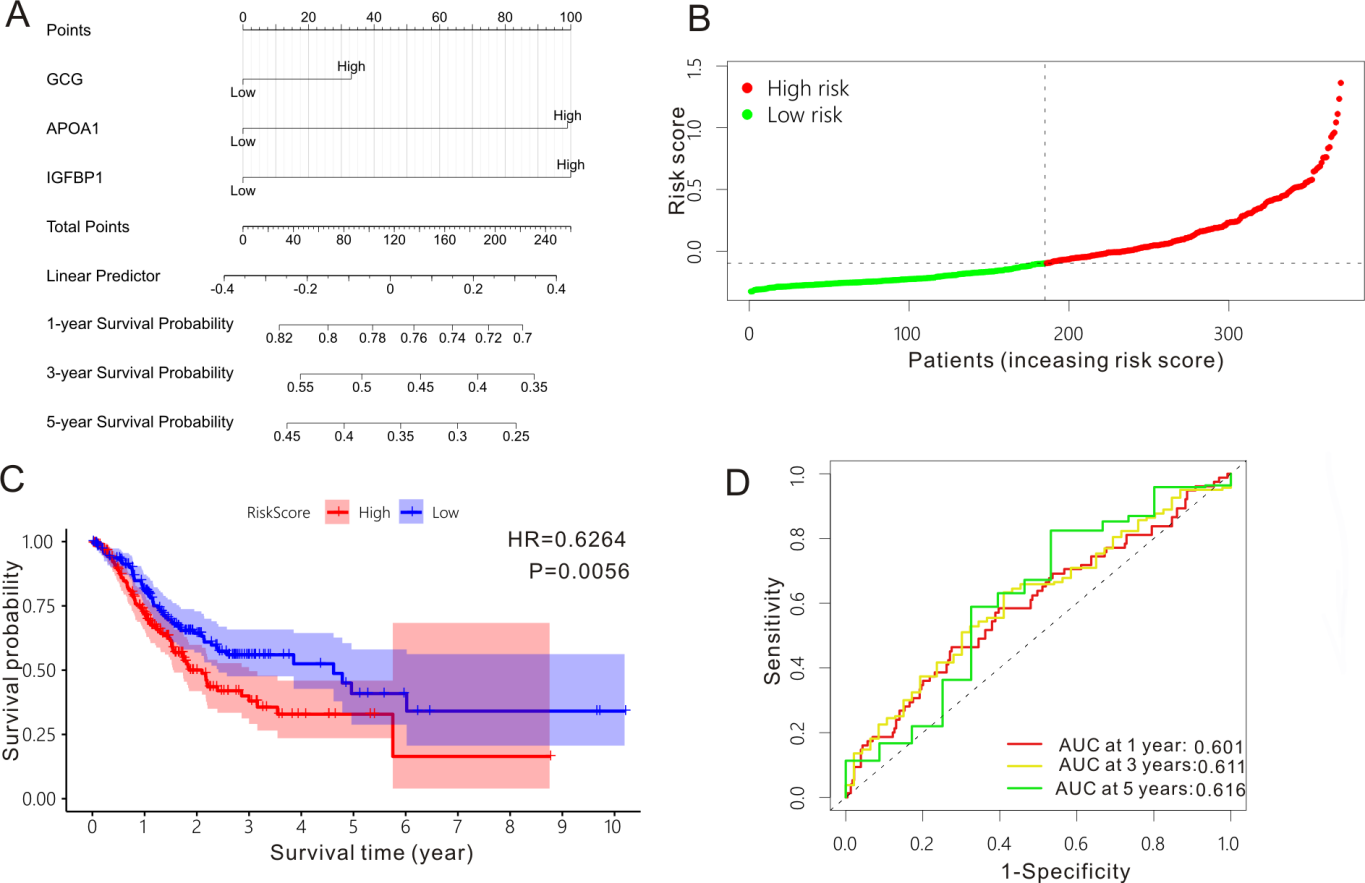
Survival analysis and predictive performance of *H. pylori*-related nomogram. (**A)** *H. pylori*-related nomogram. (**B)** Kaplan-Meier curve of gastric cancer individuals stratified by low risk and high risk. (**C)** Ranking of risk scores of gastric cancer subjects from TCGA-STAD data set. (**D)** Time-dependent ROC curves of the *H. pylori*-related nomogram.

Based on the TCGA-STAD data set, we divided the GC cases into *H. pylori* group (*N* = 20) and non-*H*. *pylori* group (*N* = 157). A fold change >2 and a false discovery rate <0.05 are considered to be significant, and we identified 4,667 significant genes between *H. pylori* group and non-*H*. *pylori* group and 4,465 genes are downregulated and 202 genes are upregulated. The top 20 significant genes between *H. pylori* group and non-*H*. *pylori* group are listed in Fig. 1A. Cox proportional hazard model incorporating three *H. pylori*-related genes (GCG, APOA, and IGFBP1), constructed by least absolute shrinkage and selection operator (LASSO) Cox (Fig. 1B and C) and multivariate Cox regression (Fig. 1D), was performed to predict the survival outcomes for GC individuals. The three *H. pylori*-related genes all possessed good predictive power for the overall survival of GC individuals (Fig. 1E). Then, we constructed a prognostic nomogram based on the three *H*. *pylori*-related genes for GC subjects (Fig. 2A) and divided these GC individuals into low-risk group and high-risk group (Fig. 2B). Survival analysis of the *H. pylori*-related nomogram demonstrated that GC subjects in low-risk group exhibited much better overall survival compared to those cases in high-risk group (*P* = 0.0056, HR = 0.6264, 95%CI: 0.4499–0.8721, Fig. 2C). Time-dependent receiver operating characteristic (Td-ROC) analysis (Fig. 2D) was utilized to comprehensively assess the predictive power of the three-gene nomogram, and the predictive performance was 0.601, 0.611, and 0.616 for 1-year overall survival, 3-year overall survival, and 5-year overall survival, respectively.

Enrichment analysis was carried out to pick out the potential biological processes between the *H. pylori* group and non-*H*. *pylori* group, and we picked out 4,667 differentially expressed genes (DEGs) with |log2 (fold change) |> 1 and FDR < 0.05 from TCGA-STAD data set. The results from Gene Ontology (GO) analysis (Fig. S1A and B) demonstrated that the differential genes between the *H. pylori* group and non-*H*. *pylori* group are mainly involved in the regulation of lipoprotein lipase activity, reverse cholesterol transport, regulation of triglyceride catabolic process, protein-lipid complex, endoplasmic reticulum lumen, steroid binding, receptor-ligand activity, and signaling receptor activator activity. Kyoto Encyclopedia of Genes and Genomes (KEGG) analysis (Fig. S1C) revealed that the differential genes mainly take part in cholesterol metabolism, vitamin digestion and absorption, fat digestion and absorption, PPAR signaling pathway, neuroactive ligand-receptor interaction, lipid and atherosclerosis, and cAMP signaling pathway. In brief, the differential genes between the *H. pylori* group and non-*H*. *pylori* group are mainly involved in key signals related to metabolism, such as cholesterol, vitamin, and fat metabolism, which indicates that *H. pylori* might promote the progression of GC partly via the regulation of metabolic pathways.

### Relationship between *H. pylori* infection and gut microbiota

A total of 97 subjects with GC were initially screened for possible recruitment. Of these, 46 cases with GC were excluded from this research due to our criteria for defining study participants. The clinical analysis presented here pertains to fecal samples from a total of 51 subjects. Among them, 25 cases of GC infected with *H. pylori* were assigned to the *H. pylori* group based on the ^13^C-urea breath test, and 26 cases of GC not infected with *H. pylori* were assigned to the non-*H*. *pylori* group. There was no difference in age and gender between the *H. pylori* group and non-*H. pylori* group.

**Fig 3:**
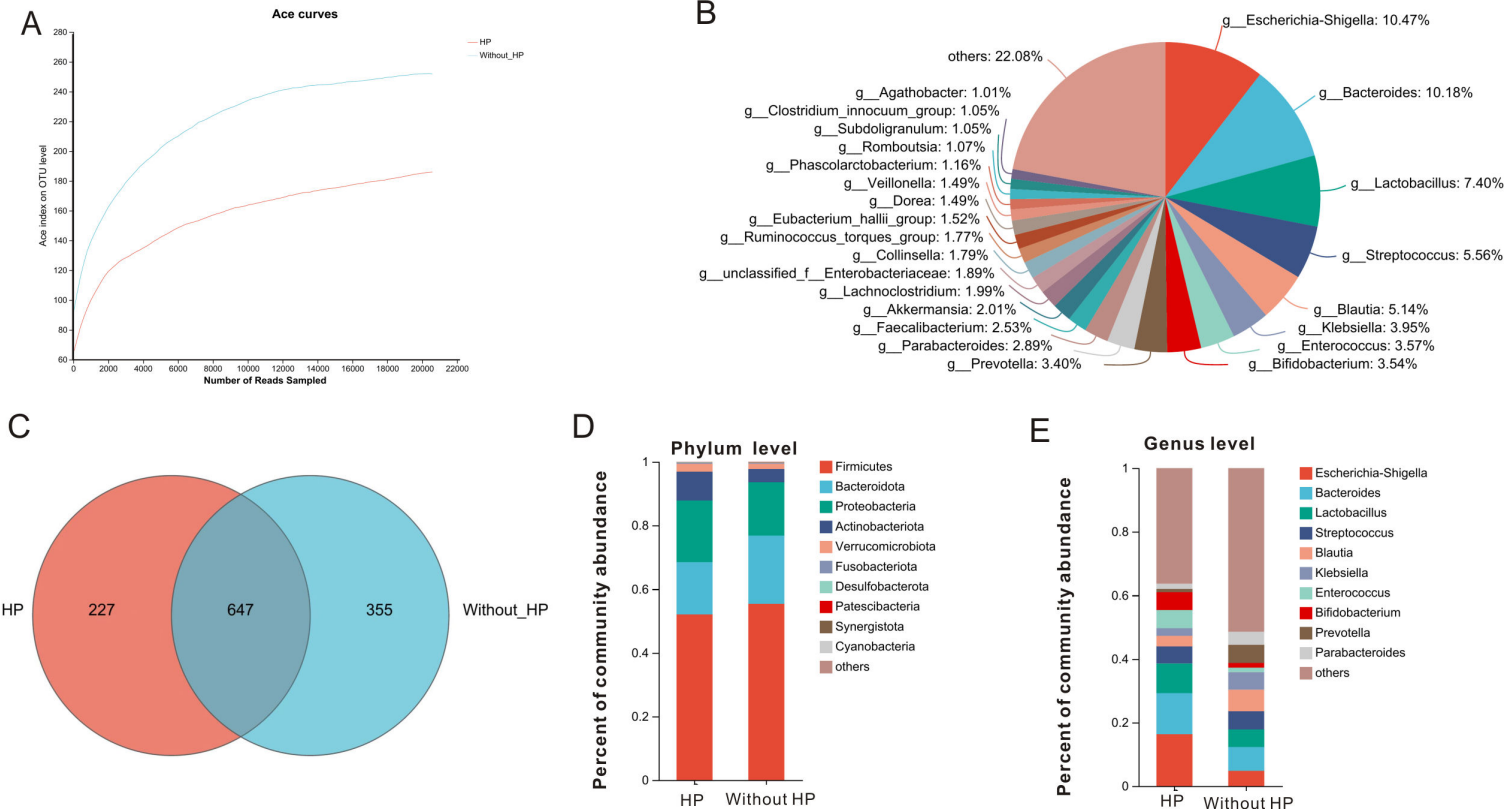
Altered fecal microbiota in gastric cancer subjects with *H. pylori* compared to cases without *H. pylori*. (**A)** Rarefaction curve. (**B)** Composition of fecal species of 51 cases of gastric cancer individuals at the genus level. (**C)** Venn plot. (**D)** Comparison of fecal microbiota between *H. pylori* group and non-*H*. *pylori* group at the phylum level. (**E)** Comparison of fecal microbiota between *H. pylori* group and non-*H*. *pylori* group at the genus level.

The rarefaction plot (Fig. 3A) is shown for *H. pylori* group and non-*H*. *pylori* group, indicating a reasonable sequencing depth of this analysis. The overall microbial community of GC subjects is shown in Fig. 3B. The 16S rRNA sequencing assay generated 1,229 operational taxonomic units (OTUs) at a 97% similarity level (Fig. 3C). Among them, 227 OTUs are unique to GC cases with *H. pylori*, and 355 OTUs are unique to GC cases without *H. pylori*. The abundance of gut microbes between the *H. pylori* group and non-*H*. *pylori* group at the phylum level exhibited significant differences. The abundances of *Firmicutes* and *Bacteroidota* were richer in GC cases without *H. pylori*, while the abundances of *Proteobacteria*, *Actinobacteriota*, and *Verrucomicrobiota* were decreased in GC cases without *H. pylori* compared to cases with *H. pylori* (Fig. 3D). At the genus level (Fig. 3E), the abundances of *Escherichia-Shigella, Bacteroides, Enterococcus, and Lactobacillus* were richer in GC cases with *H. pylori*, while the abundances of *Klebsiella* and *Blautia* were decreased in GC cases with *H. pylori* compared to cases without *H. pylori*. As *H. pylori* is a major human pathogenic bacterium located in gastric mucosa, the detection rate of *H. pylori* via 16S rRNA sequencing technique from fecal samples is usually very low (17). This may be the reason why *H. pylori* was not detected as one of the dominant bacteria in the present analysis.

**Fig 4:**
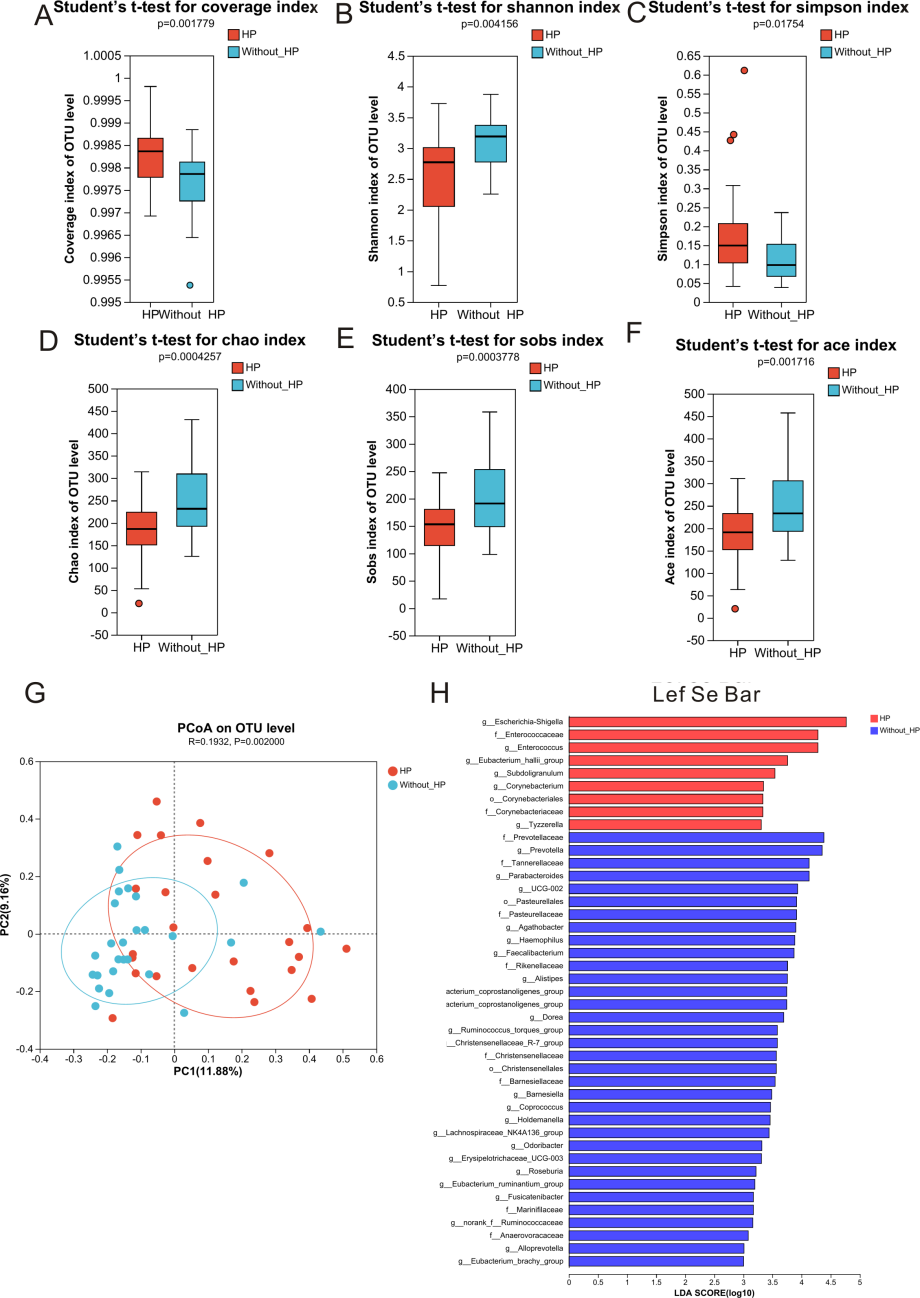
The alpha and beta diversity of fecal microbiota between gastric cancer cases with *H. pylori* and cases without *H. pylori*. There exists significant alpha diversity between gastric cancer cases with *H. pylori* and without *H. pylori.* (**A)** Goods coverage, (**B)** Shannon index, (**C)** Simpson index, (**D)** Chao index, (**E)** Sobs index, and (**F)** ACE index. (**G)** Beta diversity presented by PCoA between *H. pylori* group and non-*H. pylori* group was statistically significant. (**H)** LEfSe analysis shows that there was a huge difference in species diversity between the *H. pylori* group and non-*H*. *pylori* group.

For alpha diversity analysis, richness and evenness of microbial community as represented by Goods coverage (*P* = 0.00178, Fig. 4A), Shannon (*P* = 0.004156, Fig. 4B), Simpson (*P* = 0.01754, Fig. 4C), Chao (*P* = 0.0004257, Fig. 4D), Sobs index (*P* = 0.0003778, Fig. 4E), and ACE index (*P* = 0.01754, Fig. 4F) demonstrated significant differences between the *H. pylori* group and *non-H. pylori* group. Alpha diversity analysis indicated that species richness was significantly decreased in GC cases with *H. pylori* compared to cases without *H. pylori*. We found that PCo1 is 11.88% and PCo2 is 9.16% (Fig. 4G) according to principal-coordinate analysis (PCoA) and the beta diversity presented by PCoA between the *H. pylori* group and non-*H. pylori* group was statistically significant (*P* = 0.002). Linear discriminant analysis effect size (LEfSe) analysis is a comparative method utilized to identify significant biomarkers that may explain differential phenotypes. LEfSe analysis showed that there was a huge difference in species diversity between the *H. pylori* group and non-*H. pylori* group as detected by the Wilcoxon rank-sum test (Fig. 4H). A total of 43 species at the genus level were identified in *H. pylori* group and non-*H. pylori* group when the cutoff value of linear discriminant analysis (LDA) was set at 3. Nine gut bacteria were enriched in the *H. pylori* group, including *Escherchia-Shigella, Enterococcaceae, Enterococcus, Eubacterium_hallii_*group*, Subdoligranulum*, and 34 species at the genus level were enriched in the non-*H*. *pylori* group, including *Prevotellaceae, Prevotella, Tannerellaceae, Parebacteroides*, and *UCG-002*.

### Correlation between *H. pylori* and metabolomic profiles

**Fig 5:**
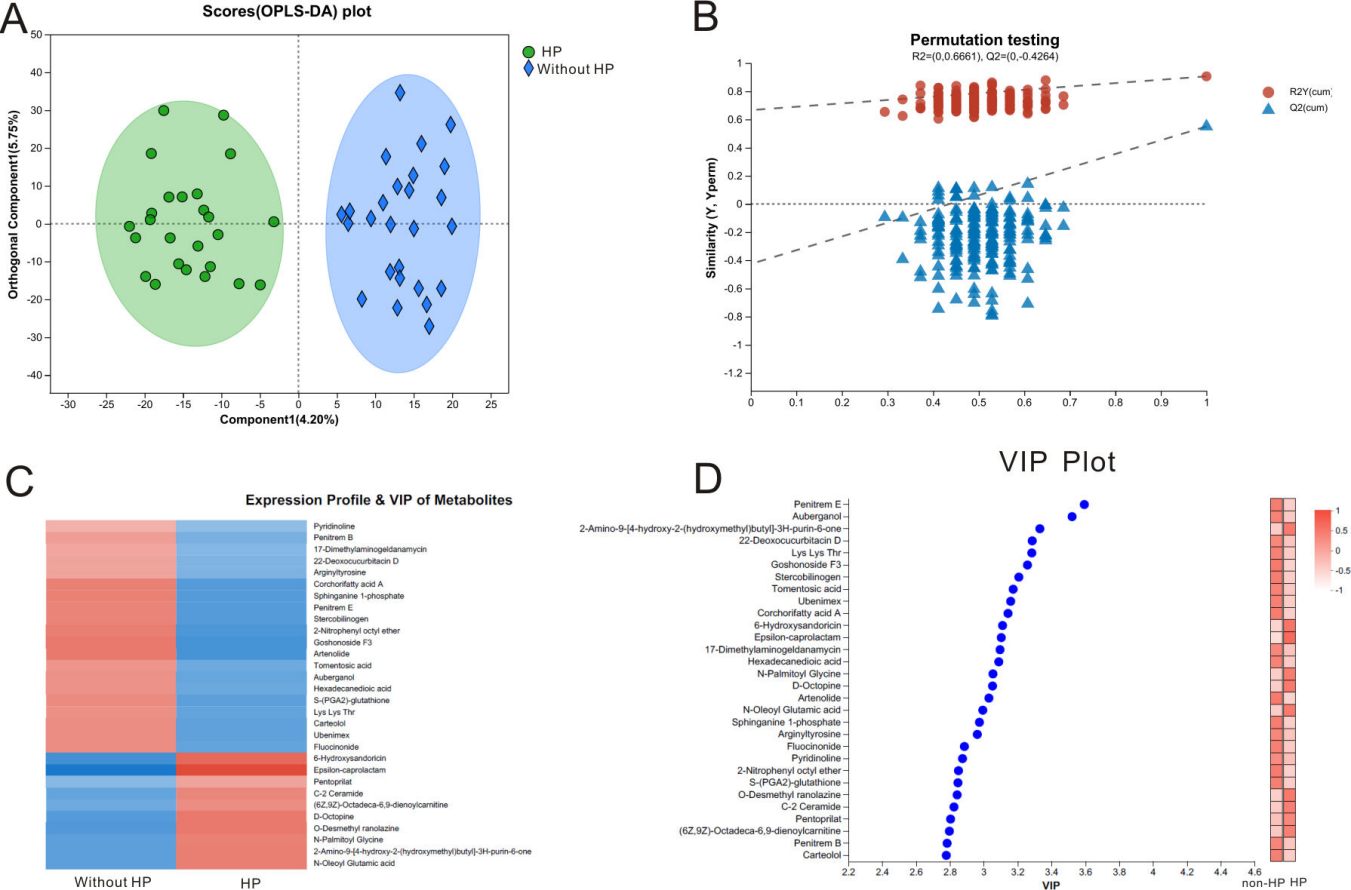
The fecal metabolites of gastric cancer subjects with *H. pylori* are significantly different from those without *H. pylori*. (**A)** OPLS-DA plot shows that fecal samples from gastric cancer were separated into two clusters. (**B)** The test for OPLS-DA model indicates the OPLS-DA model is suitable for this analysis. (**C)** Comparison of fecal metabolites between *H. pylori* group and non-*H*. *pylori* group. (**D)** VIP plot of the significant fecal metabolites between the *H. pylori* group and non-*H*. *pylori* group.

Untargeted metabolome analysis of 51 subjects with GC was carried out to explore the significant metabolites associated with *H. pylori* infection among GC individuals, and 295 fecal metabolites were quantified using ultra high performance liquid chromatography-electrospray tandem mass spectrometry (UHPLC-MS/MS). The fecal samples in the *H. pylori* group and non-*H*. *pylori* group were vividly separated into two distinct parts (component 1: 4.2%, orthogonal component 1: 5.75%) and good repeatability in each group, which was shown in the orthogonal least partial squares discriminant analysis (OPLS-DA) score curve (Fig. 5A). The test for the PLS-DA demonstrated that the value of *R2* (*R2*: 0–0.6661) was greater than the value of *Q2* (0–−0.4246), suggesting that the PLS-DA model for the present metabolome analysis was valid (Fig. 5B). There were 30 significant metabolites with relatively differential abundance between the *H. pylori* and non-*H*. *pylori* groups (Fig. 5C). Then, we used the variable importance in projection (VIP) analysis to determine which significant metabolites are upregulated in GC subjects with *H. pylori* infection. Among the 31 kinds of significant fecal metabolites, the relative abundance of 20 metabolites was upregulated in *H. pylori* group than in the non-*H*. *pylori* group. We found that the abundances of penitrem E, auberganol, stercobilinogen, and lys thr were upregulated in *H. pylori* group, while the abundances of 2-amino-9, D-octopine, N-palmitoyl glycine, and N-oleoyl glutamic acid were significantly upregulated in non-*H*. *pylori* group (Fig. 5D).

**Fig 6:**
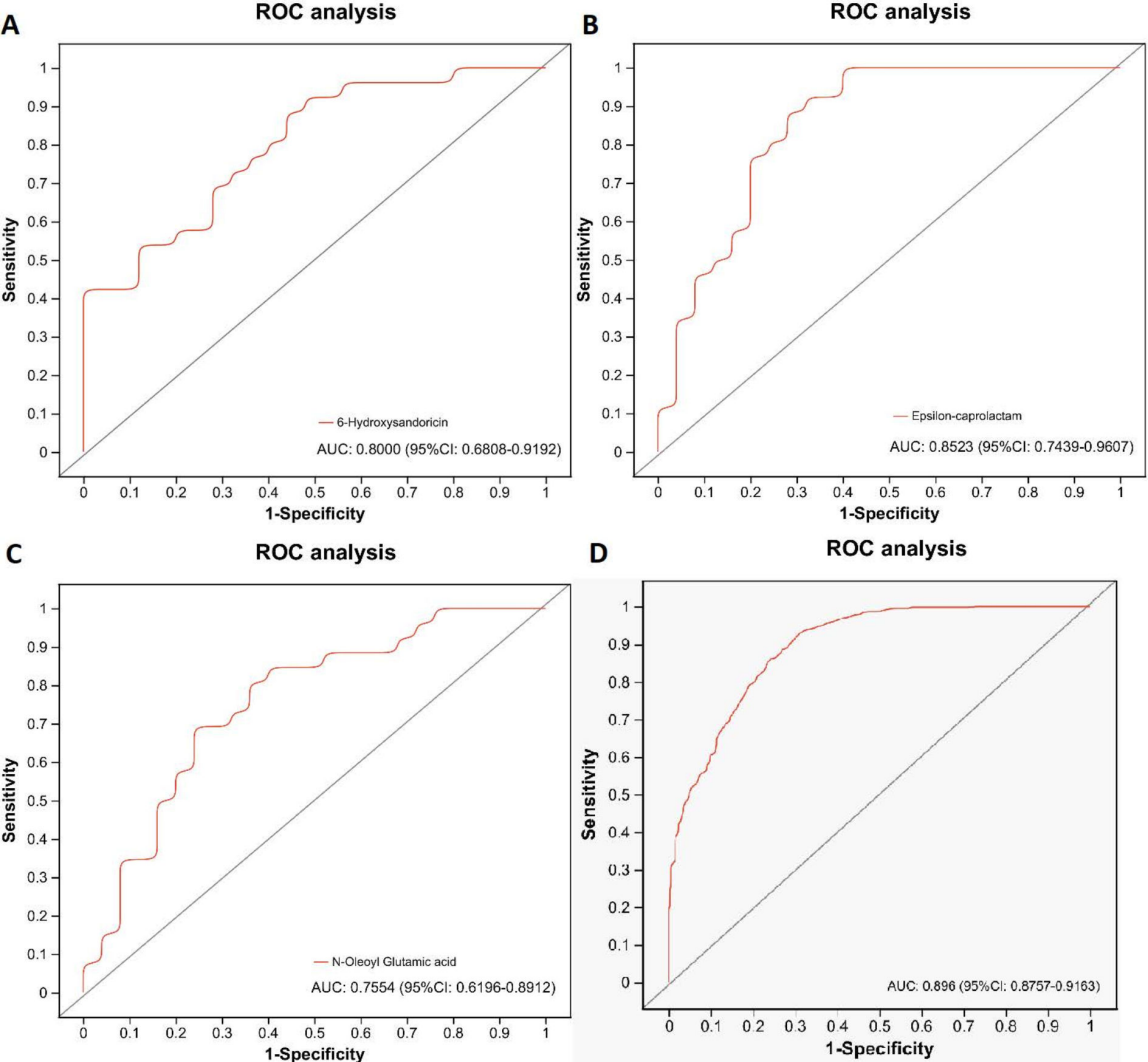
ROC analysis of significant metabolites for discriminating gastric cancer subjects with *H. pylori* from cases without *H. pylori* infection. (**A)** 6-Hydroxysandoricin, (**B)** Epsilon-caprolactam, (**C)** N-oleoyl glutamic acid, and (**D)** combination.

To diagnose GC individuals who are infected with *H. pylori* from the perspective of metabolic biomarkers, we chose the significant fecal metabolites for the subsequent receiver operating characteristic (ROC) analysis. Fecal metabolites with area under the curve (AUC) less than 0.75 were excluded from candidate biomarkers. Therefore, three fecal metabolites (6-hydroxysandoricin, epsilon-caprolactam, N-oleoyl glutamic acid) were finally selected for discriminating GC subjects with *H. pylori* from cases without *H. pylori* infection. The corresponding AUCs of fecal metabolites (Fig. 6A through C) were 0.80, 0.8523, and 0.7554, respectively. The combination of the three fecal metabolites (Fig. 6D) generates nice diagnostic power as measured by AUC of 0.896 (95% CI: 0.8757–0.9153), indicating the three metabolites (6-hydroxysandoricin, epsilon-caprolactam, N-oleoyl glutamic acid) are significantly correlated with *H. pylori* infection in gastric cancer and could serve as candidate markers for discriminating GC subjects with *H. pylori* from cases without *H. pylori* infection.

### Enrichment analysis of differential metabolites

KEGG enrichment analysis was executed to determine the important metabolic signal pathways associated with the differential fecal metabolites in the *H. pylori* group and non-*H*. *pylori group*. As shown in Fig. S2, the differential fecal metabolites are mainly involved in the biosynthesis of alkaloids derived from ornithine and lysine, tropane, piperdine and pyridine alkaloid biosynthesis, melanogenesis, lysine degradation, axon regeneration, phenylalanine, tyrosine and tryptophan biosynthesis. Moreover, the cAMP signaling pathway, retinol metabolism, and NF-kappa B signaling pathway are also enriched. In summary, enrichment analysis reveals that the differential fecal metabolites between the *H. pylori* group and non-*H*. *pylori* group are involved in significant metabolic pathways, which are related to the progression of GC.

### Association between discriminative metabolites and gut microbes

The composition and diversity of the fecal microbiota were remarkably different between the *H. pylori* group and non-*H*. *pylori* group, alterations in the metabolic substance might be partly affected by intestinal microbiota. Hence, we conducted the Spearman correlation analysis to determine the potential relationship between gut microbiota and fecal metabolites in individuals with GC. The results from correlation analysis showed that there exists a significant correlation between the fecal metabolites and gut bacterial strains (Fig. S3). Among these, the abundance of g_*Eubacterium_Eligens*_group is positively 1-cyclohexyl-3-[2-(4-elthxyphoxy) ethyl]urea, stercobilin, 1-[2-(1,3-benzodioxol-5-yl)-2-hydroxypropyl]-3-cyclohexylurea, while negatively correlated with palmitoylcarnitine and (2R,3Z)-phycocyanobilin. Similarly, *g_Lachnospiracwae_FCS020_*group was reversely correlated with linoleoyl ethanolamide, lentialexin, 3-hydroxybutanoic acid, 6-hydroxybutanoic acid, styrene, aliocholic acid, hydroxypropionic acid, palmitoylcarnitine, N1, N12-diacetylspermine, while positively correlated with 1-cyclohexyl-3-[2-(4-elthxyphoxy)ethyl]urea, stercobilin, 1-[2-(1,3-benzodioxol-5-yl)-2-hydroxypropyl]-3-cyclohexylurea. Moreover, the levels of 1-cyclohexyl-3-[2-(4-elthxyphoxy)ethyl]urea, stercobilin, 1-[2-(1,3-benzodioxol-5-yl)-2-hydroxypropyl]-3-cyclohexylurea were also positively correlated with the abundance of *g_UCG_003, g_Norank_f_Ruminococcaceae*, and *g_Fusicatenibacter*.

## DISCUSSION

This is the first clinical research to systematically illustrate the difference between GC individuals with *H. pylori* and GC individuals without *H. pylori* from the perspective of multi-omics. From bioinformatic analysis, we identified three significant genes (GCG, APOA1, and IGFBP1) associated with the infection of *H. pylori*, and the survival nomogram based on the *H. pylori*-related genes exhibited good predictive power for survival outcome among GC subjects. Next, results from 16S rRNA sequencing based on 51 cases of fecal samples showed a significantly decreased microbial richness and evenness in GC subjects with *H. pylori* compared to GC cases without *H. pylori*. The relative abundances of *Escherichia-Shigella, Bacteroides, Lactobacillus*, and *Streptococcus* were significantly altered in GC individuals with *H. pylori*. Finally, untargeted metabolome analysis showed that the majority of the metabolites, such as N-oleoyl histidine, tetraphyllin B, mauritine A, and 8-deoxylactucin, exhibited increased levels in the GC individuals without *H. pylori* compared to the cases with *H. pylori*. The combination of the significant metabolites exhibits nice diagnostic power for the discrimination of GC cases with *H. pylori* from GC cases without *H. pylori*. More importantly, correlation analysis between gut microbiota and fecal metabolites signifies the complex interaction in GC.

Our genetic study related to *H. pylori* shares several similarities with the three previous researches but also shares a little difference with them. Soutto et al. (18) unveil a significant protective function of TFF1 in alleviating *H. pylori*-mediated inflammation, which is an obvious hallmark of gastric carcinogenesis. The absence of TFF1 expression might be a critical step in *H. pylori*-mediated gastric tumorigenesis. Kim et al. (19) evaluated the activation of nuclear factor kappa B in the *H. pylori*-infected gastric epithelium of mice, and *H. pylori* infection was reported to stimulate the production of proinflammatory factors via nuclear factor kappa B in mice, highlighting the significant role of *H. pylori* in the initiation gastric inflammation. Lu et al. (20) carried out methylation-specific PCR analysis to determine the methylation status of the RUNX3 in atrophic gastritis and gastric carcinoma with *H. pylori*, and they concluded that stepwise methylation of RUNX3 promoter mediated by *H. pylori* infection contributes to the initiation and progression of gastric carcinoma. Our genetic research used comprehensive bioinformatic analysis to identify the differentially expressed genes between *H. pylori* group and non-*H*. *pylori* group, and then used LASSO Cox to further select the prognostic genes in GC. Hence, we identified three key genes closely associated with *H. pylori* infection, including GCG, APOA1, and IGFBP1. The survival nomogram constructed by the three *H*. *pylori*-related genes could well differentiate high-risk GC individuals from low-risk GC individuals.

*H. pylori* has been regarded as a definite carcinogenic bacteria for gastric carcinoma (21). A better understanding of the relationship between *H. pylori* infection and gut microbiota in gastric lesions is quite significant for the assessment of the overall benefits of eradication therapy among GC subjects with *H. pylori* infection. Gao et al. (22) performed deep sequencing of 16S rRNA in fecal samples from 47 subjects with benign gastric lesions and discovered that the microbiota alterations of *Bacteroidetes, Proteobacteria*, and *Firmicutes* are involved in the progression of *H. pylori*-related benign gastric lesion. Gantuya et al. (23) used the 16S rRNA technique to identify the different microbiota between 40 chronic gastritis subjects with *H. pylori* and 11 cases without *H. pylori*, and they found that *Haemophilus parainfluenzae* and *Streptococcus* sp. are significant pathogenic bacteria for chronic gastritis subjects without *H. pylori*. A population-based cohort (24) from China demonstrated that successful eradication of *H. pylori* could restore gut microbiota to a similar status as found in GC cases without *H. pylori*, indicating that eradication of *H. pylori* in GC will not exacerbate microbial dysbiosis. In our analysis, we also employed the 16S rRNA technique to detect the different gut microbiota between the *H. pylori* group (*N* = 25) and non-*H*. *pylori* group (*N* = 26) to reveal the possible relationship between *H. pylori* infection and gut microbiota among GC individuals. Similar to their findings, we also notice the significant difference in *bacteroidetes* and *Streptococcus* at the genus level between the *H. pylori* group and non-*H*. *pylori* group.

The four most significant gut microbiota in GC individuals with *H. pylori* are *Escherichia-Shigella, Bacteroides, Enterococcus, and Lactobacillus*. This is the first clinical research to reveal the differential gut microbiota between *H. pylori* and non-*H*. *pylori* among GC individuals, and *Escherichia-Shigella* is the most significant bacteria at the genus level. Previous studies reported that the abundance of *Escherichia-Shigella* was significantly increased in GC tissues compared to the normal gastric tissues (25–27), indicating that this *Escherichia-Shigella* might play an important role in the progression of GC. Another study has proved that *Escherichia-Shigella* is the dominant bacteria in the gut microbiome, and its abundance is positively correlated with the level of TNF-α in patients with tuberculous meningitis (28). As for *Bacteroides* and *Enterococcus*, they are the common organisms of the gastric microbiota in GC and also interact with *H. pylori* to contribute to the progression of GC. A recent study pointed out that *Lactobacillus* was higher in GC tissues compared to normal tissues (29), and Sonveaux et al. demonstrated that *Lactobacillus* could produce some harmful metabolites that could be used for tumor growth (30). Hence, based on the results of 16S rRNA sequencing, we put forward that *H. pylori* interacts with *Escherichia-Shigella, Bacteroides, Enterococcus*, and *Lactobacillus* to accelerate the progression of GC.

Comprehensive metabolomics is a novel technique in medical research that would gain insights into a better understanding of the role of *H. pylori* in GC. Human metabolites are easily affected by the human genome, while the bacterial genome also occupies a significant role in the biosynthesis of human metabolites. However, the influence of gut microbes on metabolite biosynthesis in GC individuals with *H. pylori* remains unclear. Nagata et al. (31) pointed out that *H. pylori* metabolites derived from cholesterol aggravate gastric inflammation via the activation of C-type lectin receptors. Moreover, another report (32) demonstrated that *H. pylori* consumes cholesterol in gastric glands to suppress γ-interferon signaling, thus allowing itself to escape the host immune response. Liu et al. (33) conducted the untargeted metabolomics from 25 cases of blood samples (*H. pylori*: 6, non-*H*. *pylori*: 19) and found that the citric acid and carbohydrate metabolism might be upregulated after *H. pylori* infection in GC. Except for the limited cases of GC, Liu et al.’s work did not assess the interplay between fecal metabolites and gut microbes in GC individuals with *H. pylori*. Hence, our study, for the first time, explored the difference in the fecal metabolites between GC cases with *H. pylori* and GC cases without *H. pylori*. We found that the three metabolites (6-hydroxysandoricin, epsilon-caprolactam, N-oleoyl glutamic acid) are significantly correlated with *H. pylori* infection in gastric cancer and could serve as candidate markers for discriminating GC subjects with *H. pylori* from cases without *H. pylori* infection.

Gastric microbes are easily influenced by some clinical factors, such as gastric surgery and antibiotic use. A strength of this research is that our results were not confounded by such clinical factors as we excluded GC subjects with gastric surgery or antibiotic use. Moreover, we only included GC patients who did not receive anti-*H*. *pylori* therapy. While two limitations still existed in our research. A limitation of our study is that we collected fecal samples from 51 GC individuals who underwent endoscopy examination, and the small sample size (*N* = 51) limited the feasibility of further exploring the correlation between gut microbes or fecal metabolites and important features, such as tumor-node-metastasis (TNM) stage, tumor size, and survival outcomes. Although we identified some gut microbes and fecal metabolites closely associated with *H. pylori* infection in GC subjects, how these factors contribute to the progression of GC at the cellular level still needs further validation via biochemistry experiments.

### Conclusion

This is the first clinical research to investigate the difference between GC patients with *H. pylori* and GC patients without *H. pylori* via multi-omics analysis. We established a survival nomogram at the transcription level based on four *H. pylori*-related genes in gastric carcinoma. 16S rRNA sequencing along with untargeted metabolomics demonstrated decreased microbial diversity and metabolic dysregulation in gastric carcinoma individuals with *H. pylori* infection, which provides a crucial basis for a better understanding of eradication therapy among the GC population.

## MATERIALS AND METHODS

### Comprehensive bioinformatic analysis

TCGA was used to download TCGA-STAD RNA sequencing data. A total of 370 subjects with GC, including 20 cases of GC samples with *H. pylori*, were obtained from the data set. The gene expression between *H. pylori* and non-*H*. *pylori* groups was as differentiated by the “limma” package. Genes with a fold change of more than 2 and a false discovery rate of less than 0.05 are defined as significantly differentially expressed genes between *H. pylori* and non-*H*. *pylori* groups. The differentially expressed genes related to *H. pylori* infection were further selected into the LASSO Cox analysis via the glmnet R package. An *H. pylori*-related nomogram integrating both the *H. pylori*-related DEGs, and significant prognostic genes was constructed based on the TCGA-STAD data set. The risk score of each GC patient could be calculated based on the medium value of the *H. pylori*-related nomogram, and these GC cases were divided into high-risk and low-risk groups. Finally, we utilized the “ClusterProfile” package to perform GO enrichment analyses along with the KEGG pathway of co-expression genes in GC.

### Collection of clinical samples with GC

We performed a clinical study of participants prospectively included from October 2022 to April 2023 from Wuhan Union Hospital. The inclusion criteria of this research were as follows: (i) subjects were pathologically diagnosed with GC; (ii) subjects received upper endoscopy examination and underwent gastric biopsy; (iii) all subjects underwent *H. pylori* detection via ^13^C-urea breath test; and (iv) subjects were willing to provide their stool samples for our research. By contrast, the exclusion criteria of this research were as follows: (i) subjects who underwent gastric surgery; (ii) subjects who experienced *H. pylori* eradication therapy; (iii) subjects who are receiving anti-infectious therapy; and (iv) subjects with age over 18 years. A total of 97 subjects with GC were initially screened for possible recruitment, while only 51 GC patients met the inclusion criteria and agreed to provide stool samples, which were collected and frozen at −80°C. The study plan of this multi-omics research was checked and approved by the Institutional Review Boards of Wuhan Union Hospital (No. 2021-S066), and all the subjects with GC gave their written informed consent. Moreover, all the procedures of this multi-omics research were conducted under the principles of the Helsinki Declaration.

### 16s rRNA sequencing for fecal microbiota analysis

The total microbial genomic DNA of 51 stool samples was extracted using the PF Mag-Bind Stool DNA Kit (Omega Bio-tek, GA, USA). The V3–V4 region of the microbial 16S rRNA gene was amplified with primer pairs 806R and 338F. All the analyses of the fecal microbiota were finished based on the platform of the Majorbio Cloud (https://cloud.majorbio.com). Shannon index, Chao richness, Goods coverage, Sobs index, and ACE index were measured to assess the alpha diversity between *H. pylori* and non-*H*. *pylori* groups. PCoA was performed to evaluate the similarity among the gut microbe communities in different GC individuals using the Vegan v2.4.3 package. The LEfSe was employed to pick out the significantly abundant taxa of bacteria (LDA score >3) between *H. pylori* and non-*H*. *pylori* groups.

### Untargeted metabolomics

Fecal samples following methanol-assisted protein precipitation and analyzed by liquid chromatography-mass spectrometry (LC-MS) using the UHPLC System (Agilent Technologies, Santa Clara, CA, USA). The Q Exactive mass spectrometer operates in positive and negative polarity mode, the spray voltage was 3.2 kV, and the data acquisition was completed with the data dependent acquisition. The pretreatment of LC/MS raw data in this research was conducted by Progenesis QI software (Waters Corporation, Milford, USA). The metabolites from 51 cases of fecal samples were identified by searching databases, including Majorbio Database, Metlin database (https://metlin.scripps.edu/), and HMDB database (https://hmdb.ca/). Principal component analysis and OPLS-DA were completed by the R package “ropls”(version 1.6.2). The fecal metabolites with *P* value less than 0.05 were regarded as significant metabolites between *H. pylori* and non-*H*. *pylori* groups. Differential fecal metabolites between *H. pylori* and non-*H*. *pylori* groups were further selected into their biochemical pathways through enrichment analysis based on KEGG database (http://www. genome.jp/kegg/).

### Statistical analysis

All statistical analyses between *H. pylori* and non-*H*. *pylori* groups were performed using SPSS 19.0 software (SPSS Inc., Chicago, IL, USA). R software (version 3.4.1) was utilized for drawing figures. Data are shown as means ± standard deviation for the continuous indexes, and the difference between *H. pylori* and non-*H*. *pylori* groups is detected by Student’s *t*-test or non-parametric test. While categorical indexes are shown as values with percentages, the difference between *H. pylori* and non-*H*. *pylori* groups was detected by chi-square test or Fisher’s exact test. Survival analysis based on the risk value of three *H*. *pylori*-related genes was performed to assess the prognostic role of the *H. pylori*-related nomogram. The ROC curves were plotted to select metabolic biomarkers for *H. pylori*-positive GC, and AUC was used to measure the power for discriminating *H. pylori*-positive GC from *H. pylori*-negative GC. Statistical significance was regarded as *P* value < 0.05 on both sides.

## Data Availability

The original data analyzed in the present study are available from the corresponding author upon reasonable request.

## SUPPLEMENTAL MATERIAL

Supplemental material mbio.01531-23-s0001.pdf Figures S1 to S3.

ASM does not own the copyrights to Supplemental Material that may be linked to, or accessed through, an article. The authors have granted ASM a non-exclusive, world-wide license to publish the Supplemental Material files. Please contact the corresponding author directly for reuse.
