# Supplementary figures and images for: Comparison of Helicobacter pylori positive and negative gastric cancer via multi-omics analysis
Source: mBio. 2023 Oct 17;14(6):e01531-23. doi: 10.1128/mbio.01531-23 (PMC10746152; doi:10.1128/mbio.01531-23)

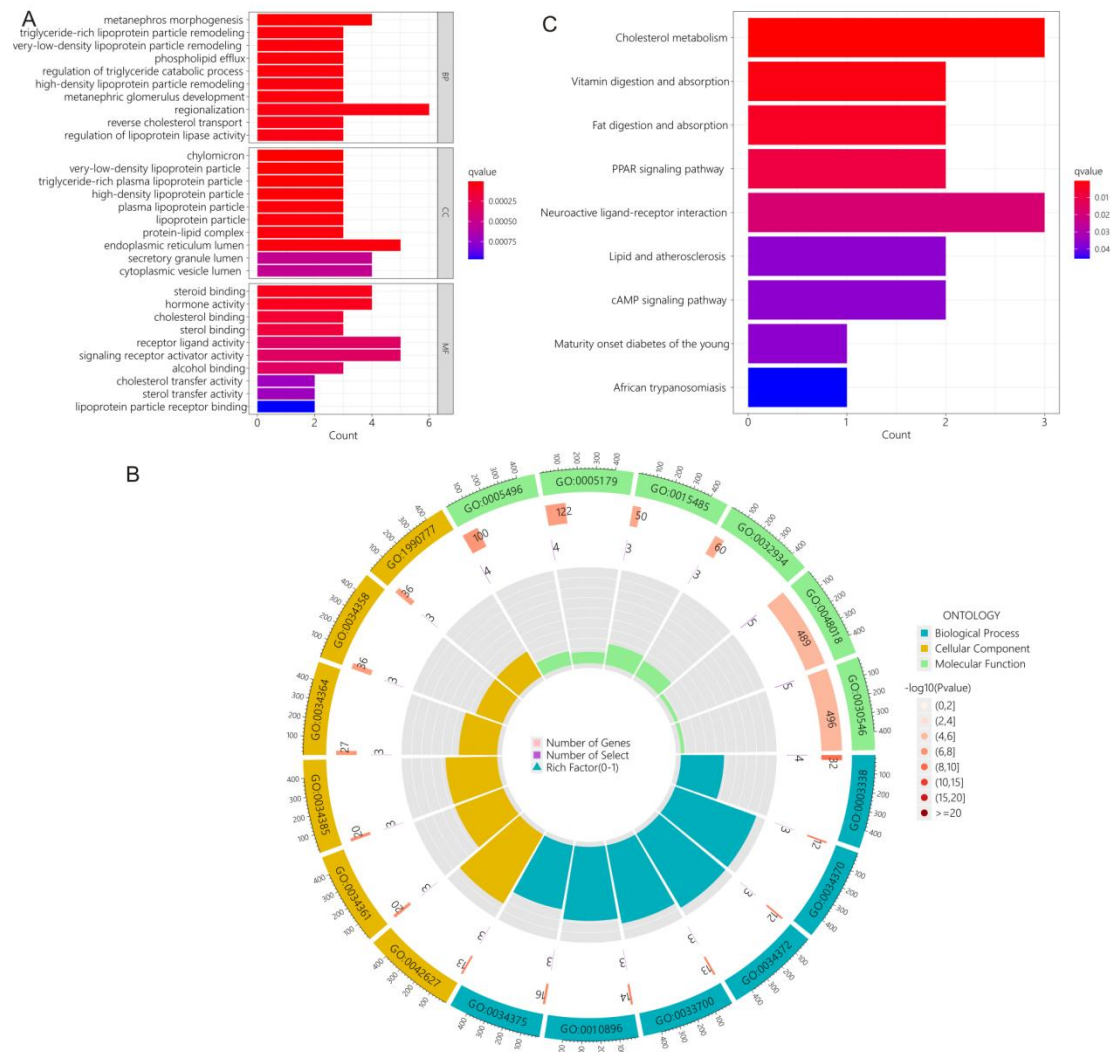



strains.

Supplement: Supplemental material — Figures S1 to S3. [file mbio.01531-23-s0001.pdf]
